# Pathways of Economic Inequalities in Maternal and Child Health in Urban India: A Decomposition Analysis

**DOI:** 10.1371/journal.pone.0058573

**Published:** 2013-03-29

**Authors:** Srinivas Goli, Riddhi Doshi, Arokiasamy Perianayagam

**Affiliations:** 1 Development Studies, International Institute for Population Science, Deonar, Mumbai, India; 2 Parkview Health, Fort Wayne, Indiana, United States of America; MIT, United States of America

## Abstract

**Background/Objective:**

Children and women comprise vulnerable populations in terms of health and are gravely affected by the impact of economic inequalities through multi-dimensional channels. Urban areas are believed to have better socioeconomic and maternal and child health indicators than rural areas. This perception leads to the implementation of health policies ignorant of intra-urban health inequalities. Therefore, the objective of this study is to explain the pathways of economic inequalities in maternal and child health indicators among the urban population of India.

**Methods:**

Using data from the third wave of the National Family Health Survey (NFHS, 2005–06), this study calculated relative contribution of socioeconomic factors to inequalities in key maternal and child health indicators such as antenatal check-ups (ANCs), institutional deliveries, proportion of children with complete immunization, proportion of underweight children, and Infant Mortality Rate (IMR). Along with regular CI estimates, this study applied widely used regression-based Inequality Decomposition model proposed by Wagstaff and colleagues.

**Results:**

The CI estimates show considerable economic inequalities in women with less than 3 ANCs (CI  = −0.3501), institutional delivery (CI  = −0.3214), children without fully immunization (CI  = −0.18340), underweight children (CI  = −0.19420), and infant deaths (CI  = −0.15596). Results of the decomposition model reveal that illiteracy among women and her partner, poor economic status, and mass media exposure are the critical factors contributing to economic inequalities in maternal and child health indicators. The residuals in all the decomposition models are very less; this implies that the above mentioned factors explained maximum inequalities in maternal and child health of urban population in India.

**Conclusion:**

Findings suggest that illiteracy among women and her partner, poor economic status, and mass media exposure are the critical pathways through which economic factors operate on inequalities in maternal and child health outcomes in urban India.

## Introduction

There has been an exponential rise in urban population around the globe during the last century [1], [2]. The global urban population almost quadrupled from 732 million in 1950 to an estimated 3.2 billion in 2005. The proportion of urban population increased from just 13% in 1900 to 49% in 2005 [3]. The latest United Nations population projections indicate that the global urban population will rise to 4.9 billion in 2030, amounting to 60% of the global population. In developing countries, the proportion of urban population is expected to increase to 57% by 2025 [4]. As per provisional reports from the 2011 Census of India, almost one third (377 million) of India's population resides in urban areas [5]. This number is projected to rise to 432 million by 2021 [6].

The process of urbanization is regarded as an important factor associated with socioeconomic growth, resulting in elimination of the traditional socioeconomic hierarchy [7], [8], [9]. However, the process of urbanization in developing countries is not identical to that of the developed countries. With the adaptation of Liberalization, Privatization and Globalization (LPG) policies, the socioeconomic structure of urban areas in the developing countries has changed considerably. The urban-centric globalization in the developing world not only boosts overall economic growth, but also increases the economic inequalities [10], [11], [12], [13], [14], [15], [16], [2], [17], [18], .

Urbanization in India is widening the gaps within the existing economic order, instead of turning into more “generative” for a new economic order [21]. Urban growth is associated with increase in the proportion of urban poor residing in slums. The total slum population in large cities (with a population of hundred thousand and above) of India is as high as 37.3 million. Moreover, a large proportion of slum population in India belongs to lower economic group [22]. Recent evidence on changing income distribution within the context of the economic growth and the rising average household income in India has fuelled a debate on the possible impact of urban growth on health inequalities. In India, the speed of urban growth has outpaced the development of essential infrastructure that is required for safe and healthy. In addition to the socioeconomic and demographic differences, private sector has a greater presence in urban areas, leading to higher cost of health care [21], [17], [23], [24], [25].

Existing global urban health literature recognizes the existence of intra-urban economic inequalities in health. The appropriate assessment of the health needs of urban population is crucial to planning and management of urban healthcare [26], [16], [27], [19], [28]. Previous studies on urban health disparity in India mostly focused on differentials in means of health indicators of different socioeconomic groups by slum and non-slum population. Furthermore, very few studies have carried out regression analyses [29], [30], [31], [32], [33], [34], . However, the health policies for an urbanizing society cannot be based on average health status alone. There is an urgent need to acknowledge and assess the intra-urban socioeconomic disparities. The urban poor in India have different living and health conditions than their richer counterparts. There is a crucial need for setting priorities and developing targeted strategies to address economic inequalities and its pathways of influencing poor health status in urban population of India. Moreover, women and children are more vulnerable when it comes to economic inequalities in health and are the first to be affected by economic inequalities. Thus, maternal and child health have been given more attention in Millennium Development Goals (MDGs).

India, with its current infant mortality rate of 47 per 1000 live births and maternal mortality ratio of 212 per 100000 live births, may not be able to achieve the goal 4 and 5 of the MDGs, respectively [37]. One of the most important factors responsible for India's inability to achieve the MDGs by 2015 is the existing economic inequalities in access of maternal and child care services. Therefore, the two principal aims of this study are to assess the economic inequalities in maternal and child health indicators of urban India; and calculate relative contribution of different socioeconomic factors to total economic inequalities in maternal and child health indicators.

## Methods

### Data

Analyses was performed based on data from the third wave of the National Family Health Survey (NFHS-3) conducted in 2005–06, by the International Institute for Population Sciences, Mumbai under the stewardship of the Ministry of Health and Family Welfare (MoHFW), Government of India. NFHS-3 survey is conducted in tandem with the global Demographic Health Survey (DHS). It is a nationwide representative sample survey of 109,041 households, 124,385 women and 74,369 men of age group 15–54 years. The survey collected information on fertility, mortality, morbidity, maternal and child health, with representative samples from all the 29 states of India, which comprises more than 99 percent of the national population [38]. Moreover, the sample of households, women and children for each economic group of urban India are adequate to carry out any robust estimates and draw appropriate conclusions and their results are generalizable to the urban Indian context (Appendix S1).

The survey adopted a three-stage sample design for urban areas. In urban areas, wards were selected during the first stage, Census Enumeration Blocks (CEB) containing approximately 150/200 households were selected during the second stage, and the required numbers of households were selected for the third stage using systematic sampling technique. The households and eligible female informant response rates were consistently above 99%. For further details on sampling, please refer to the report published by IIPS and Macro-Internationals, 2007 [38].

### Ethics Statement

Ethical approval for this survey was obtained from the International Institute for Population Sciences, Mumbai, India. The informed consent was obtained from all the respondents before conducting interviews. A standard consent form approved by the ethics review committee was read to the respondent in the respondent's native language. Once the respondent agreed to participate in the survey, the interviewer confirmed this consent and signed on the form acknowledging that the respondent had been read the form, had understood the study and agreed to participate. The information collected in the survey purely used for research works and never the name and place of the respondents have been disclosed to a third person.

### Variables

The outcome variables for this study comprise of the following critical maternal and child health indicators: antenatal check-ups (ANCs), institutional delivery, underweight children, children with full immunization and Infant Mortality Rate (IMR). For the purpose of assessment of economic inequalities in maternal and child health indicators, these indicators were categorized into two groups: disadvantageous and advantageous groups respectively (e.g. less than 3 ANCs/three or more than three ANCs; Not an institutional delivery/Institutional delivery; children underweight/children not underweight; children without complete immunization/children with complete immunization; Infant death/not an infant death).

In the estimation of economic inequalities in maternal and child health indicators, the scale of economic status (wealth quintile) of the household is the key variable. Economic status is based on the mean of the household wealth status, which is based on 33 assets and housing characteristics. Each household asset is assigned a weight (factor score) generated through Principle Component Analysis (PCA), and the resulting assets scores are standardized in relation to the normal distribution with a mean of zero and standard deviation of one. The sample was divided into five wealth quintiles.

Predictor variables for the inequality decomposition analyses included key socio-economic and demographic variables to calculate the contributions and determine pathways of economic inequality in maternal and child health indicators of urban population. The socioeconomic and demographic variables were also dichotomized into disadvantageous and advantageous groups (e.g. poor/non poor economic status, illiterate/literate, SCs STs/Other, Muslims/other religion, not working/working, no mass media exposure/mass media exposure) to perform the inequality decomposition analyses. There is empirical evidence which indicates that SCs STs and Muslim religion groups are in a disadvantageous position in terms of maternal and health child status compared to the general population [29], [30], [31], [32], [33], [34], [35], [25], [36], [38].

### Methods of Analyses

The methods of analyses take into consideration two issues evident in existing health inequality literature. Firstly, a long-standing concern in the study of health inequality is whether or not all inequalities should be measured or solely those showing some systematic association with indicators of socioeconomic standing should be measured [39], [40], [41], [42]. With this under consideration, only key maternal and child health indicators were selected to measure economic inequalities in the urban population of India. Secondly, the household economic status is a strong determinant of the education, social and health beliefs, family building ways, demographic behavior, media exposure, risk of illness and purchasing power of health care services [43], [44]. Thus, the economic inequalities are operating through pathways of various social and demographic characteristics. Based upon this perspective, this study examined the social and demographic pathways of economic inequalities in health of the urban population in India with particular focus on maternal and child health.

Economic inequalities in maternal and child health are calculated by using Concentration Index (CI), a method proposed by Wagstaff and Colleagues [45]. However, economic status cannot explain inequality in a health variable completely. The economic inequality in a health variable often operates through a number of intermediate variables called ‘pathways’ [46], [47]. Therefore, CI is decomposed to explore the pathways that lead to the economic inequalities in health of urban population and thereby evaluating the estimated proportional contribution of socioeconomic and demographic factors on health inequalities (45, 47).

In this study, decomposition analysis is separately carried out for five key health variables 1) less than 3 ante-natal check-ups, 2) not an institutional delivery, 3) children without complete immunization, 4) underweight children, and 5) infant deaths as dependent variables. The decomposition of health inequalities in urban population of India is carried out in following steps described by Wagstaff and colleagues [45] and Donnell and colleagues [47]:

1. Coefficients of the predictor variables (*β_k_*) are estimated by regressing the health variables through linear regression model for its socioeconomic predictors.
2. Means of the health variable and each of its predictors (µ and 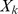) are estimated.
3. Concentration indices for the health variable and its predictors (C and 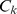) are estimated using equation (1) along with generalized concentration index of error term 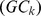 where, 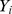
and µ are the value of the predictors for the i^th^ individual and the predictors mean, respectively.
4. Absolute contribution of each predictor is estimated by multiplying the health variable elasticity with respect to the predictor and its concentration index – 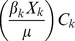

5. Percentage contribution of each predictor is calculated by dividing its absolute contribution by the concentration index of health variable – 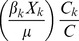

The mathematical equations used in the decomposition analyses are following:
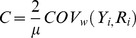
(1)where 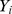 is the health status of the *i* ^th^ individual and *R_i_* is the fractional rank of the *i* ^th^ individual (for weighted data) in terms of *the index of household economic status*; µ is the (weighted) unconditional mean of the health variable of the sample and 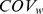 denotes the weighted covariance. It reveals the concentration of inequalities among the subgroup of population.

The equation above estimates the CI which is twice the (weighted) covariance of the health variables, and a person's relative rank in terms of economic status, divided by the variable mean. The individuals are ranked in ascending order of their household living standard in order to find out the cumulative fraction, for example women with less than 3 ante-natal check-ups, by their economic status. The weights are used to adjust for the design effect of sample survey data. The value of CI lies between −1 and +1 where negative value implies concentration of outcome variable among disadvantageous groups and positive value implies concentration among advantageous groups. The zero value of concentration index implies no inequality (45).

Wagstaff et al., (2003) [46] has proposed following linear regression model that links health variable of interest, Y, to a set of k health determinants, *X_k_*. This linear regression is estimated separately for each of the health variable i.e. less than ante-natal check-ups, not an institutional delivery, children not fully immunized, underweight children, and infant deaths by linking them to the socioeconomic predictors. The predictors for different health variables are not exactly the same. The predictors of different health variables are selected based on our review of existing literature [42], [43], [38], [40], [48], [49], [23], [41], [35], [50], [51].



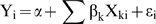
(2)where ε is an error term. Given the relationship between *Y_i_* and 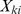 in equation, the concentration index for Y (C) can be written as:
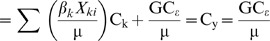
(3)


The above equation shows that C comprises of two components. The first is the deterministic or ‘explained’ component. This is equal to a weighted sum of the concentration indices of the regressors, where the weights are elasticities [elasticity is a unit-free measure of (partial) association, i.e. the percent change in the dependent variable (health variables) associated with a percent change in the predictor variables], 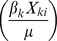
of Y with respect to each 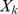.The second is a residual or ‘unexplained’ component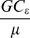, where GC is the generalized concentration index. The explained component reflects that proportion of the inequalities in the dependant variable (health variable) which are explained by the systematic variation in the selected predictor's i.e.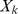, the unexplained component reflects that part of inequalities which could not be explained by the selected predictors across socioeconomic groups. Stata version 10.1 (Stata crop LP, College Station, Texas, USA) and Microsoft excel program were used to perform this statistical analyses.

## Results

### Social and demographic disparities by economic groups

In India, social and demographic characteristics of the population residing within urban areas differ significantly by wealth status (Table 1). For example, the highest proportion of women with no education is among the poorest wealth quintile (64%) followed by poorer wealth quintile (52%); in contrast, only 30% and 17% of the richer and richest groups respectively have no education. Similar pattern is also observed in case of uneducated partners by household wealth quintile. The poorest-richest gap in no education is 47% among women and 43% among their partners. The proportion of SCs/STs and Muslim women is also more among the poorest and poorer wealth quintiles compared to other wealth quintiles. The proportion of people having no mass media exposure is considerably high among the lower economic groups compared to the higher economic groups. About 55% of the poorest economic group has no mass media exposure compared to only 1.5% in the richest economic group. The poorest-richest gap in no mass media exposure is 53.5%. Unemployed women are also more likely to be poor than their counterpart. About 42% of women are not working in poorest than only 7.2% of richest wealth quintile. Similarly, women in the poorest wealth quintile have disadvantageous demographic indicators. For instance, birth order three and more is substantially greater among women with poorest wealth quintile (42%) compared to richest (7%). The poorest-richest gap in birth order 3+ children is 34%. These results strengthen the argument that the wealth status has a greater effect on the socioeconomic and demographic conditions, which further influence health status of the urban population in India.

**Table 1:** Social and demographic disparities by economic groups (wealth quintile) in urban India, NFHS-3, 2005–06.

States	Percentage women aged 15–49 with no education		Percentage of women with no educated partners		Proportion of SC/ST population		Proportion of Muslim religion population		Percentage of women aged 15–49 not have mass media exposure		Percentage of women with birth order 3+		Percentage of women aged 15–49 not working women	
Wealth Quintile	Urban	Total	Urban	Total	Urban	Total	Urban	Total	Urban	Total	Urban	Total	Urban	Total
Poorest	63.9	60.2	57.5	56.3	40	47.7	24.2	16.5	54.6	61.7	41.6	42.2	41.1	55.5
Poorer	51.8	50.5	44.6	35.4	31	33.4	26.2	16.8	34.8	43.8	34.5	32.2	31.9	44.0
Middle	41.5	41.1	28.6	24.2	27	25.6	25.6	18.2	21.2	28.1	25.5	23.1	23.5	34.8
Richer	29.6	29.6	13.5	9.7	25	21.1	26.3	18.8	9.1	12.8	18.0	15.7	17.9	23.1
Richest	16.7	17.3	2.7	2.6	13	12.7	15.7	14.7	1.5	2.9	7.2	7.2	14.7	15.6
**Poorest-** **Richest gap**	**47.2**	**42.9**	**54.8**	**53.7**	**27**	**35**	**8.5**	**1.8**	**53.1**	**58.8**	**34.4**	**35**	**26.4**	**39.9**

### Health disparities by economic groups

The health disparities in key maternal and child health indicators by wealth quintile for the urban population of India are displayed in Table 2. The results indicate that the proportion of women with less than three antenatal checkups (ANCs) and who had non-institutional deliveries are more likely to belong to the poorest wealth quintile (98% and 74%) as compared to women belonging to the richest wealth quintile (48% and 13%). The poorest-richest gap in these two indicators is considerably higher i.e. 50% and 61%, respectively for <3 ANCs and non-institutional delivery. The same pattern is apparent in the case of child immunization and underweight children. The proportion of underweight children and children without complete immunization are highest in poorest wealth quintile (61% and 73%) than their richest counterparts (26% and 28%). The richest-poorest gap in these two indicators is 35% and 44% respectively, for underweight and children without complete immunization. IMR is the highest in the poorest wealth quintile (90 per 1000 live births) and lowest in the richest wealth quintile (35 per 1000 live births). The richest-poorest gap in infant deaths is as high as 55 per 1000 live births. For all the maternal and child health indicators considered for this study, the assessment of health disparities by economic groups reveals that poorest and poorer wealth quintile are at a disadvantageous position compared to other economic groups.

**Table 2:** Heath disparities by economic groups of urban India, NFHS-3, 2005–06.

States	Percentage who not had at least three Antenatal Checkups (ANCs)^1^		Percentage of births not delivered in any health facility^2^		Percentage of children (aged 0–4 years) underweight^3^		Children (aged 12–23 months) not fully immunized4		Infant Mortality Rate (IMR)^5^	
Wealth Quintile	Urban	Total	Urban	Total	Urban	Total	Urban	Total	Urban	Total
Poorest	97.9	87.6	74.0	87.0	60.6	61.3	72.8	75.6	90.0	64.8
Poorer	95.6	84.4	64.0	76.7	56.2	54.5	66.5	66.8	71.0	62.2
Middle	87.2	79.5	49.0	61.0	48.3	47.1	56.0	53.0	59.0	49.8
Richer	71.0	75.7	34.0	42.0	41.3	39.6	44.4	44.7	50.0	46.2
Richest	48.3	72.9	13.0	16.0	25.8	25.4	28.3	29.0	34.7	27.4
**Poorest-** **Richest gap**	**49.6**	**14.7**	**61**	**71**	**34.8**	**35.9**	**44.5**	**46.6**	**55.3**	**37.4**

Note: 1 & 2. ANCs estimated based on the recent birth to ever-married women in the three years preceding the survey.

3. Underweight estimates are based on a new international reference population recommended by World Health Organization (WHO) in April 2006 (WHO Multicenter Growth Reference Study Group, 2006). Children age group 0–5 years, whose weight-for-age is below -2SD from the median of the reference population, is classified as underweight.

4. Full immunization includes children who received BCG, measles, and three doses each of DPT and polio (excluding polio 0).

5. IMR estimated as probability of dying in age group 0 to 11 months in five years birth history of women.

### Effects and contribution of factors in economic inequalities of health

The previous sections discuss the disparities in socioeconomic, demographic and health conditions of women and children by their household economic status in urban India. However, setting health priorities based on differences in averages of health indicators can be misleading. The Urban health averages mask intra-urban inequalities when, these are disaggregated on the household economic scale. Therefore, in this section we calculate the magnitude of effects in terms of economic disparity on health inequalities measured by CIs.

**Figure 1:**
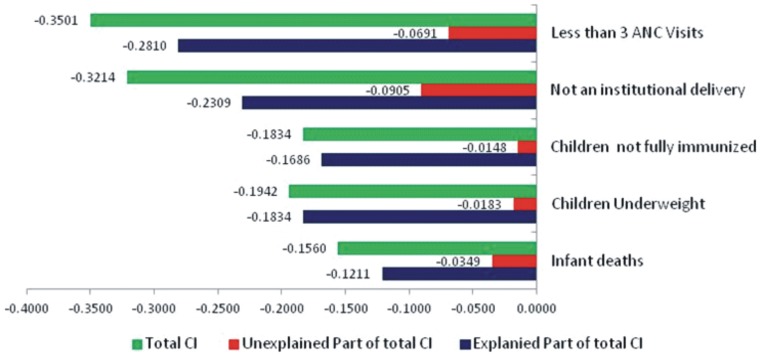
Concentration indices for selected health indicators in urban India, NFHS-3, 2005–06.

Figure 1 presents the value of CIs which are calculated for negative indicators: less than three ANC visits, not an institutional delivery, children without complete immunization, underweight children and infant deaths in the urban India. CIs are found to be negative for all the selected indicators thereby confirming that the economically weaker stratum of the population in urban India is disadvantaged in terms of maternal and child health. However, it is also observed that there is considerable difference in the magnitude of economic inequalities in the five selected maternal and child health indicators. The economic inequalities are highest for the indicator – less than three ANC visits (−0.3501). However the CIs for other indicators: not an institutional delivery, children without complete immunization, children underweight and infant deaths are also substantially high which is, −0.3214, −0.1834, −0.1942, −0.1560 respectively. Overall, CI estimates reveal that health inequalities are greater in maternal health indicators than child health indicators.

Economic inequalities influence human health through multi-dimensional channels; therefore, explaining the pathways of economic inequalities in maternal and child health of urban population in India is critical for health policy. This study investigates the pathways of urban health inequalities using inequality decomposition analyses. The results of decomposed contributions of different socioeconomic and demographic factors for maternal and child health inequalities in urban India are presented in Table 3. The results reveal that selected socioeconomic factors contribute remarkably to the inequalities in maternal and child health indicators. The decomposition of CI for women receiving less than three ANCs in urban India indicates that the seven socioeconomic predictors explained 81% (−0.2810 out of −0.3501) of total inequality in ANC coverage. The remaining 19% could not be explained by the predictors under consideration for this study (Appendix S2). The results of the relative proportional contributions of socioeconomic predictors demonstrate that around 42% (p<.05) of the total inequalities in ANC coverage are explained by women's illiteracy followed by poor economic status (36%, p<0.05).

**Table 3:** Contribution of socio-economic factors to health inequalities among selected health variables in urban India, NFHS-3, 2005–06.

Predictors	% Contribution to CI (95% CI bootstrap)				
	<3 Anti- Natal checkups	Not an institutional delivery	Children not fully immunized	Children underweight	Infant deaths
Male child	-	-	0.33 (−0.034, 0.1)	−0.05 (−005, −0.097)	−0.80 (−0.003, −0.164)
Poor economic status	**36.15** (16.05, 56.25)	**19.5** (7.02, 31.9)	**26.39** (12.8, 40)	**51.40** (23.2, 79.7)	**21.22** (11, 31.44)
Woman/Mother's illiteracy	**42.09** (20.09, 64.10)	**26.6** (11.6, 41.5)	**31.15** (13.1, 49.17)	**24.30** (9.02, 39.6)	**34.69** (14.9, 54.48)
Husband/Father's illiteracy	**12.27** (5.88, 18.64)	**6.5** (1.2, 11.9)	**12.14** (5.03, 19.26)	**11.22** (4.6, 17.84)	**14.75** (6.58, 22.92)
Belonging to SCs/STs households	2.48 (0.04, 4.9)	4.1 (0.9, 7.4)	5.61 (−0.09, 11.19)	1.81 (0.05, 3.56)	3.45 (1.04, 5.86)
Belonging to Muslim religion households	1.52 (0.09, 2.94)	1.1 (−0.02, 2.3)	**4.82** (0.4, 9.2)	1.37 (−0.04, 2.8)	−3.82 (−1.008, −6.64))
Birth order 3+	-	-	**10.63** (2.3, 18.9)	**4.97** (1.3, 8.64)	**16.20** (4.5, 28)
No mass media exposure	**6.53** (1.5, 11.56)	**4.6** (0.4, 8.9)	**8.94** (1.6, 16.2)	4.97 (0.98, 8.95)	**12.05** (3.9, 20.21)
Not working	−1.03 (−4.09, 2.02)	2.3 (−0.1, 4.65)	-	-	2.25 (−1.5, 6.01)
<3 Anti- Natal checkups	-	**35.2** (16.4, 54)	-	-	-
Not a Institutional delivery	-	-	-	-	-
**Total**	**100**	**100**	**100**	**100**	**100**

Note: 1) (−) indicates that the variables is either not a relevant predictor or marginal effect of the to the health variable is not statistically significant at p<0.05; therefore not included in the estimation of decomposed contributions.

2) % contribution figures in **bold** indicates significant contributions p<0.05 of bootstrap analyses.

3) The figures may be affected by round-up.

Table 3 also presents the estimates of proportional contribution of the selected socioeconomic factors to inequalities in non-institutional delivery. The results indicate that inequalities in institutional deliveries are largely contributed by not having three and more ANCs (35%, p<0.05), women's illiteracy (27%, p<0.05) and poor economic status of household (19%, p<0.05). The eight selected factors together explain 72% of total inequality in not an institutional delivery (Appendix S3). In case of inequalities in children without complete immunization, the results reveal that mother's illiteracy is the highest contributor (31%, p<0.05), followed by the poor economic status (26%, p<0.05), father's illiteracy (12%, p<0.05) and birth order greater than three (11%, p<0.05). The selected factors together explain 92% of total inequalities (i.e. −16860 out of −0.18340) in children without complete immunization (Appendix S4). Similarly, the decomposition results for inequalities in underweight children (Appendix S5) indicate that selected factors together explains 95% of total inequalities (i.e. −19420 out of −0.18340). The results of proportional contribution of individual factors reveal that poor economic status is the largest contributor to the children being underweight (51%, p<0.05), followed by mother's illiteracy (24%, p<0.05) and father's illiteracy (11%, p<0.05).

Inequality decomposition model results for infant deaths (Appendix S6) reveal that nine socioeconomic predictor variables explain 78% of the inequalities (−0.1211 out of −0.15596). The remaining 22% constituted the unexplained residual which could not be explained by the selected predictors. The measure of proportional contribution to socioeconomic covariates indicated that 35% (p<0.05) of inequality in infant deaths is explained by illiteracy of mother, followed by poor economic status of household (21%, p<0.05) and paternal illiteracy (14%, p<0.05).

The decomposition outcomes indicate that most of the inequalities are explained by the selected socioeconomic factors, that is, wealth status, education, caste, religion, birth order, mass media exposure, working status and ANCs. For all the five maternal and child health indicators, results suggest that the levels of health inequalities are more pronounced in households with illiterate women, poor economic status, spouse illiteracy and women with no mass media exposure. However, in case of institutional deliveries, ANCs checkups are the critical contributor.

## Discussion

This paper makes a new contribution to urban health literature in India. It is unique in terms of analyses of social determinants of maternal and child health inequalities as this effort make a methodological contribution to health inequality analyses of urban India. It is generally understood that city dwellers enjoy better health than their rural counterparts; therefore, increasing urbanization has led to the continuous improvement in average health status. However, health information for urban areas is usually aggregated to provide an average of all urban residents rather than disaggregated by socioeconomic status. The average health information masks urban inequalities and very little is known about socioeconomic inequalities in health that exist within urban areas [19]. Recently, the distributional dimension of health inequality has become prominent in the global health policy agenda as researchers have come to regard average health status as an inadequate summary of a country's health performance. According to the World Health Organization, 2010 was the landmark year for urbanization and health. For the coming years, WHO has pledged to focus on health issues arising from the urban phenomenon [18].

Despite residing in urban areas, a large proportion of urban population in India is deprived of basic needs such as pucca house, safe drinking water, electricity, cooking gas, improved sanitation facilities, education and regular paid work [25], [35]. As India rapidly urbanizes, within urban areas socioeconomic disparities are rising, and health inequality among the urban population is an emerging challenge. The unprecedented rise in urbanization has threatened to reduce the supposed public health advantages of urban life in many ways -an issue social scientists are yet to consider [7], [8], [10]. In developing countries like India, urbanization poses formidable challenges for dealing with increasing urban economic disparities and consequently the rising health inequalities [15].

In order to understand these critical questions, it is important to put aside the misconceptions that have prevented the health needs of urban populations from being fully appreciated. There is an urgent need to recognize and determine the social and economic disparities among the urban population who are at different levels of development and live in different health environments. Therefore, this is a well-timed effort to analyze the extent to which economic disparities affect and in pathways along which it operates on maternal and child health inequalities in urban India. Based on quantification and decomposition of economic inequalities in different dimensions of maternal and child health within urban India, this study presents fine distinctive findings compared to earlier studies. Many of the previous studies examined urban health disparities based on rich-poor differences and CI estimates [25], [29], [30], [31], [32], [34], [35], [36]. Averages of health indicators mask within and between group disparities and CI estimates indicate only volume of inequality, moreover, both these estimates fail to explain how these inequalities are channelized. Therefore, the present study fills a critical methodological gap by using decomposition of maternal and child health inequalities to their socioeconomic factors in urban population of India.

Findings of CI estimates reveal considerable economic inequalities in maternal and child health indicators. The findings also suggest that the economic inequalities in maternal and child indicators are channelized through a number of socioeconomic characteristics namely in order of their importance: illiteracy among women and her partner, poor household economic status, lack of exposure to mass media, birth order belonging to SCs STs and Muslim religion. Woman illiteracy emerged as the key predictor for ANCs care, institutional delivery, children immunization and infant deaths but poor economic status emerged as a key predictor of children underweight in the urban India. Findings suggest that, in four out of five indicators, a greater proportion of economic inequalities are channelized through the illiteracy status of women. Overall, in the context of urban population of India, inequality decomposition analyses make a unique contribution in identifying critical pathways of economic inequalities in maternal and child health inequalities.

Different economic groups have their unique socioeconomic and demographic conditions, behavior and household environment, which together are likely to influence social beliefs, family planning practices, food habits, dressing, household location, household amenities, demographic behavior, health practices and health care seeking behavior. Social and health beliefs, family building strategies and health care purchasing power largely control the utilization of health services. Further, socioeconomic conditions, health care and demographic behavior predict the health outcomes. However, poor household environment itself strongly determines the chance of getting contact with illness and utilization of health care services.

From the implication point of view, the study brings out crucial suggestions: first, public health and social policy initiatives and programmes aimed at reducing social disparity and income-related inequality in health should be targeted at specific dimensions of health for specific populations for example illiteracy of women in terms of no ANCs care and poor economic status in terms of underweight children. Second, India needs to adopt the dual strategy, of strengthening the existing social safety nets to protect socially and economically disadvantaged population and concurrently applying, health policy interventions for urban areas focusing ideally on both health averages and inequalities. Finally, this study demonstrates that obtaining equity in terms of maternal and child health status for the urban population of different economic groups in India may not seem achievable in the near future, unless the quality of urbanization and equity of distribution of the urban resources are ensured. A serious effort must be made to remove the socioeconomic dispossession, thereby reducing the health disparities in order to building healthy and sustainable cities in urban India. Healthy urbanization programmes should generate new resources and stimulate action to iron out urban health inequity. Therefore, achieving health equity for India's urban children remains a critical challenge of recently proposed national urban health mission.

## Supporting Information

Appendix S1 **Sample distribution households, women and children by economic groups in urban India.**
(DOCX)

Appendix S2 **Effects and contribution of predictor variables based on decomposition analysis for less than Anti- Natal Checkups (ANCs) in Urban India.**
(DOCX)

Appendix S3 **Effects and contribution of predictor variables based on decomposition analysis for not institutional delivery in Urban India.**
(DOCX)

Appendix S4 **Effects and contribution of predictor variables based on decomposition analysis for children not fully immunized in urban India.**
(DOCX)

Appendix S5 **Effects and contribution of predictor variables based on decomposition analysis for child underweight in urban India.**
(DOCX)

Appendix S6 **Effects and contribution of predictor variables based on decomposition analysis for Infant deaths in urban India.**
(DOCX)
