# Supplementary material for: Pathways of Economic Inequalities in Maternal and Child Health in Urban India: A Decomposition Analysis
Source: PLoS One. 2013 Mar 29;8(3):e58573. doi: 10.1371/journal.pone.0058573 (PMC3612074; doi:10.1371/journal.pone.0058573)
Supplement: Appendix S1 — Sample distribution households, women and children by economic groups in urban India. (DOCX) [file pone.0058573.s001.docx]

**Appendix S1.** Sample distribution households, women and children by economic groups in urban India, NFHS-3, 2005-06.

| **Economic groups** | **Number of Households** | **Percent** | **Number of**  **Women** | **Percent** | **Number of**  **Children** | **Percent** |
| --- | --- | --- | --- | --- | --- | --- |
| Poorest | 14638 | 13.43 | 21718 | 17.46 | 14377 | 25.47 |
| Poorer | 16566 | 15.20 | 23616 | 18.99 | 12654 | 22.42 |
| Middle | 20947 | 19.22 | 25088 | 20.17 | 11181 | 19.81 |
| Richer | 25486 | 23.38 | 26106 | 20.99 | 10154 | 17.99 |
| Richest | 31359 | 28.77 | 27856 | 22.40 | 8072 | 14.30 |
