# Supplementary material for: Pathways of Economic Inequalities in Maternal and Child Health in Urban India: A Decomposition Analysis
Source: PLoS One. 2013 Mar 29;8(3):e58573. doi: 10.1371/journal.pone.0058573 (PMC3612074; doi:10.1371/journal.pone.0058573)
Supplement: Appendix S2 — Effects and contribution of predictor variables based on decomposition analysis for less than Anti- Natal Checkups (ANCs) in Urban India. (DOCX) [file pone.0058573.s002.docx]

**Appendix S 2.** Effects and contribution of predictor variables based on decomposition analysis for less than Anti- Natal Checkups (ANCs) in Urban India, NFHS-3, 2005-06.

| **Predictors** | **Mean** | **Marginal effect** | **CI** | **Contribution to CI** | **% contribution to CI**  **(95 % CI bootstrap)** |
| --- | --- | --- | --- | --- | --- |
| Poor economic status households | 0.1304 | 0.2139 | -0.8947 | -0.1016 | **36.15**  (16.05, 56.25) |
| Woman illiteracy | 0.2830 | 0.2439 | -0.4209 | -0.1183 | **42.09**  (20.09, 64.10) |
| Husband illiteracy | 0.1644 | 0.1030 | -0.5006 | -0.0345 | **12.27**  (5.88, 18.64) |
| Belonging to SCs/STs households | 0.2272 | 0.0497 | -0.1513 | -0.0070 | 2.48  (0.04, 4.9) |
| Belonging to Muslim religion households | 0.2181 | 0.0476 | -0.1014 | -0.0043 | 1.52  (0.09, 2.94) |
| No Mass media exposure | 0.7664 | 0.0607 | -0.0969 | -0.0183 | **6.53**  (1.5, 11.56) |
| Not working | 0.8215 | 0.0332 | 0.0262 | 0.0029 | -1.03  (-4.09, 2.02) |
| **Less than 3 ANCs** | **0.2457** |  | **-0.3501** | **-0.2810** | **100.00** |
|  |  |  | **Residual** | **-0.06914** |  |

Note: 1) % contribution figures in **bold** indicates significant contributions at p value of <0.05 of bootstrap analyses.

2) The figures may be affected by round-up.
