# Supplementary material for: Pathways of Economic Inequalities in Maternal and Child Health in Urban India: A Decomposition Analysis
Source: PLoS One. 2013 Mar 29;8(3):e58573. doi: 10.1371/journal.pone.0058573 (PMC3612074; doi:10.1371/journal.pone.0058573)
Supplement: Appendix S3 — Effects and contribution of predictor variables based on decomposition analysis for not institutional delivery in Urban India. (DOCX) [file pone.0058573.s003.docx]

**Appendix S 3.** Effects and contribution of predictor variables based on decomposition analysis for not institutional delivery in Urban India, NFHS-3, 2005-06.

| **Predictors** | **Mean** | **Marginal effect** | **CI** | **Contribution to CI** | **% contribution to CI**  **(95 % CI bootstrap)** |
| --- | --- | --- | --- | --- | --- |
| Poor economic status | 0.1304 | .147 | -0.8947 | -0.0450 | **19.5**  (7.02, 31.9) |
| Woman illiteracy | 0.283 | .196 | -0.4209 | -0.0614 | **26.6**  (11.6, 41.5) |
| Husband illiteracy | 0.1644 | .070 | -0.5006 | -0.0150 | **6.5**  (1.2, 11.9) |
| Belonging to SCs/STs households | 0.2272 | .104 | -0.1513 | -0.0094 | 4.1  (0.9, 7.4) |
| Belonging to Muslim religion households | 0.2181 | .046 | -0.1014 | -0.0027 | 1.1  (-0.02, 2.3) |
| No Mass media exposure | 0.7664 | .055 | -0.0969 | -0.0107 | **4.6**  (0.4, 8.9) |
| Not working | 0.8215 | .096 | -0.0262 | -0.0054 | 2.3  (-0.1, 4.65) |
| Less than 3 ANCs | 0.2457 | .360 | -0.3501 | -0.0813 | **35.2**  (16.4, 54) |
| **Not institutional delivery** | **0.3810** |  | **-0.3214** | -0.2309 | 100.0 |
|  |  |  | **Residual** | **-0.0905** |  |

Note: 1) % contribution figures in **bold** indicates significant contributions at p value of <0.05 of bootstrap analyses.

2) The figures may be affected by round-up.
