# Supplementary material for: Pathways of Economic Inequalities in Maternal and Child Health in Urban India: A Decomposition Analysis
Source: PLoS One. 2013 Mar 29;8(3):e58573. doi: 10.1371/journal.pone.0058573 (PMC3612074; doi:10.1371/journal.pone.0058573)
Supplement: Appendix S4 — Effects and contribution of predictor variables based on decomposition analysis for children not fully immunized in urban India. (DOCX) [file pone.0058573.s004.docx]

**Appendix S 4.** Effects and contribution of predictor variables based on decomposition analysis for children not fully immunized in urban India, NFHS-3, 2005-06.

| **Predictors** | **Mean** | **Marginal effect** | **CI** | **Contribution to CI** | **% contribution to CI**  **(95 % CI bootstrap)** |
| --- | --- | --- | --- | --- | --- |
| Male Child | 0.5315 | -0.0151 | 0.0074 | -0.0001 | 0.33  (-0.034, 0.1) |
| Poor economic status | 0.1304 | 0.1528 | -0.8696 | -0.0409 | **26.39**  (12.8, 40) |
| Mother’s illiteracy | 0.283 | 0.1931 | -0.4116 | -0.0530 | **31.15**  (13.1, 49.17) |
| Father's illiteracy | 0.1644 | 0.1013 | -0.5018 | -0.0197 | **12.14**  (5.03, 19.26) |
| Belonging to SCs/STs households | 0.2272 | 0.0933 | -0.1798 | -0.0090 | 5.61  (-0.09, 11.19) |
| Belonging to Muslim religion households | 0.2181 | 0.1362 | -0.1093 | -0.0077 | **4.82**  (0.4, 9.2) |
| Birth Order 3+ | 0.174 | 0.1476 | -0.3167 | -0.0192 | **10.63**  (2.3, 18.9) |
| No Mass media exposure | 0.7664 | 0.0885 | -0.0905 | -0.0145 | **8.94**  (1.6, 16.2) |
| **Not Fully Immunized** | **0.4241** |  | **-0.18340** | **-0.16860** | **100.00** |
|  |  |  | **Residual** | **-0.01480** |  |

Note: 1) % contribution figures in **bold** indicates significant contributions at p value of <0.05 of bootstrap analyses.

2) The figures may be affected by round-up.
