# Supplementary material for: Pathways of Economic Inequalities in Maternal and Child Health in Urban India: A Decomposition Analysis
Source: PLoS One. 2013 Mar 29;8(3):e58573. doi: 10.1371/journal.pone.0058573 (PMC3612074; doi:10.1371/journal.pone.0058573)
Supplement: Appendix S5 — Effects and contribution of predictor variables based on decomposition analysis for child underweight in urban India. (DOCX) [file pone.0058573.s005.docx]

**Appendix S 5.** Effects and contribution of predictor variables based on decomposition analysis for child underweight in urban India, NFHS-3, 2005-06.

| **Predictors** | **Mean** | **Marginal effect** | **CI** | **Contribution to CI** | **% contribution to CI**  **(95 % CI bootstrap)** |
| --- | --- | --- | --- | --- | --- |
| Male child | 0.5315 | 0.0071 | 0.0074 | 0.0001 | -0.05  (-005, -0.097) |
| Poor economic status | 0.1304 | 0.2377 | -0.8696 | -0.0904 | **51.40**  (23.2, 79.7) |
| Mother’s illiteracy | 0.283 | 0.1094 | -0.4116 | -0.0427 | **24.30**  (9.02, 39.6) |
| Father's illiteracy | 0.1644 | 0.0713 | -0.5018 | -0.0197 | **11.22**  (4.6, 17.84) |
| Belonging to SCs/STs households | 0.2272 | 0.0233 | -0.1798 | -0.0032 | 1.81  (0.05, 3.56) |
| Belonging to Muslim religion households | 0.2181 | 0.0302 | -0.1093 | -0.0024 | 1.37  (-0.04, 2.8) |
| Birth order 3+ | 0.174 | 0.0473 | -0.3167 | -0.0087 | **4.97**  (1.3, 8.64) |
| No Mass media exposure | 0.7664 | 0.0376 | -0.0905 | -0.0087 | 4.97  (0.98, 8.95) |
| **Underweight** | **0.2982** |  | **-0.19420** | **-0.18340** | **100.00** |
|  |  |  | **Residual** | **-0.01080** |  |

Note: 1) % contribution figures in **bold** indicates significant contributions at p value of <0.05 of bootstrap analyses.

2) The figures may be affected by round-up.
