# Supplementary material for: Pathways of Economic Inequalities in Maternal and Child Health in Urban India: A Decomposition Analysis
Source: PLoS One. 2013 Mar 29;8(3):e58573. doi: 10.1371/journal.pone.0058573 (PMC3612074; doi:10.1371/journal.pone.0058573)
Supplement: Appendix S6 — Effects and contribution of predictor variables based on decomposition analysis for Infant deaths in urban India. (DOCX) [file pone.0058573.s006.docx]

**Appendix S 6.** Effects and contribution of predictor variables based on decomposition analysis for Infant deaths in urban India, NFHS-3, 2005-06.

| **Predictors** | **Mean** | **Marginal effect** | **CI** | **Contribution to CI** | **% contribution to CI**  **(95 % CI bootstrap)** |
| --- | --- | --- | --- | --- | --- |
| Male child | 0.5315 | 0.0075 | 0.0096 | 0.0010 | -0.80  (-0.003, -0.164) |
| Poor economic status | 0.1304 | 0.0088 | -0.8947 | -0.0257 | **21.22**  (11, 31.44) |
| Mother’s illiteracy | 0.2830 | 0.0141 | -0.4209 | -0.0420 | **34.69**  (14.9, 54.48) |
| Father's illiteracy | 0.1644 | 0.0086 | -0.5006 | -0.0179 | **14.75**  (6.58, 22.92) |
| Belonging to SCs/STs households | 0.2272 | 0.0048 | -0.1513 | -0.0042 | 3.45  (1.04, 5.86) |
| Belonging to Muslim religion households | 0.2181 | -0.0083 | -0.1014 | 0.0046 | -3.82  (-1.008, -6.64)) |
| Birth order 3+ | 0.1740 | 0.0145 | -0.3089 | -0.0196 | **16.20**  (4.5, 28) |
| No Mass media exposure | 0.7664 | 0.0078 | -0.0969 | -0.0146 | **12.05**  (3.9, 20.21) |
| Working status | 0.8215 | -0.0050 | 0.0262 | -0.0027 | 2.25  (-1.5, 6.01) |
| Infant deaths | **.0399** |  | **-0.15596** | **-0.1211** | **100.00** |
|  |  |  | **Residual** | **-0.0349** |  |

Note: 1) % contribution figures in **bold** indicates significant contributions at p value of <0.05 of bootstrap analyses.

2) The figures may be affected by round-up.
